# A decrease in brown adipose tissue activity is associated with weight gain during chemotherapy in early breast cancer patients

**DOI:** 10.1186/s12885-020-6591-3

**Published:** 2020-02-04

**Authors:** Angeline Ginzac, Bertrand Barres, Marion Chanchou, Emilie Gadéa, Ioana Molnar, Charles Merlin, Bruno Coudert, Emilie Thivat, Xavier Durando

**Affiliations:** 1 Centre Jean PERRIN, INSERM, U1240 Imagerie Moléculaire et Stratégies Théranostiques, Université Clermont Auvergne, F-63011 Clermont-Ferrand, France; 2 Délégation Recherche Clinique & Innovation, Centre Jean PERRIN, Centre de Lutte contre le Cancer, 58 rue Montalembert, F-63011 Clermont-Ferrand, France; 3 Centre d’Investigation Clinique, UMR501, F-63001 Clermont-Ferrand, France; 4 Service de Médecine Nucléaire, Centre Jean PERRIN, Centre de Lutte contre le Cancer, 58 rue Montalembert, F-63000 Clermont-Ferrand, France; 5 CH Emile ROUX, 12 Boulevard Docteur, F-43000 le Puy en Velay, France; 6 Département d’oncologie médicale, Centre Georges-François Leclerc, 1 rue du Professeur Marion, F-21000 Dijon, France

**Keywords:** Brown adipose tissue, Energy metabolism, Weight gain, Breast cancer

## Abstract

**Background:**

A decrease in thermogenesis is suspected to be implicated in the energy expenditure reduction during breast cancer treatment. This study aimed to investigate the impact of chemotherapy on the metabolic activity of brown adipose tissue (BAT) and the link with weight variation.

**Methods:**

This was an ancillary analysis of a multicentre trial involving 109 HER2+ breast cancer patients treated with neoadjuvant chemotherapy. A centralised review of ^18^F-FDG uptake intensity (SUV_max_) in specific BAT regions (cervical and supraclavicular) was conducted on two PET-CT scans for each patient (before and after the first course of chemotherapy).

**Results:**

Overall, after one course of chemotherapy a significant decrease of 4.4% in ^18^F-FDG-uptake intensity was observed. It was not correlated to initial BMI, age or season. During chemotherapy, 10.1% (*n* = 11) of the patients lost weight (− 7.7 kg ± 3.8 kg; ie, − 9.4% ± 3.7%) and 29.4% (*n* = 32) gained weight (+ 5.1 kg ± 1.7 kg; ie, + 8.5% ± 2.6%). Among these subgroups, only the patients who had gained weight underwent a significant decrease (13.42%) in ^18^F-FDG uptake intensity (*p* = 0.042).

**Conclusion:**

This study is the first to highlight in a large cohort of patients the negative impact of chemotherapy on brown adipose tissue activity. Weight gain during chemotherapy could thus potentially be explained in part by a decrease in brown adipose tissue activity.

## Background

Excess body weight is a recognized breast cancer risk factor and also a factor of poor prognosis at diagnosis (high recurrence and mortality rates) [1, 2]. Weight gain during breast cancer treatment, in particular during chemotherapy, is also linked to poor prognosis [3–7]. The mechanisms that explain weight change are not clearly understood [8]. Weight change results from an energy imbalance, i.e. food intake versus energy expenditure [9, 10]. Nausea, vomiting or even loss of appetite for example can disturb food intake, thus affecting energy intake [9]. Energy expenditure is explored by way of resting energy expenditure (REE), physical activity and adaptive thermogenesis. BAT (brown adipose tissue) contains numerous mitochondria harbouring a particular protein, the uncoupling protein-1 (UCP-1) which confers a specific function to this tissue: heat production [11]. Thus, BAT contributes to thermogenesis [12].

BAT has received considerable attention since it is considered as a potential target to limit obesity and metabolic syndromes. Its contribution to human energy metabolism needs further investigation. BAT is known to affect energy metabolism in murine models. Indeed, Lowell et al. showed that the ablation of BAT leads to obesity [13]. Feldmann et al. drew the same conclusion, showing that UCP1 ablation led to obesity in mice [14]. Studies using fluorine-18 fluorodeoxyglucose (^18^F-FDG) positron emission tomography (PET) scans have evidenced the presence of BAT in adults [15–18]. ^18^F-FDG is currently used in oncology to mark tumours but it is not specific and has been found in high glucose metabolism organs such as BAT [19–21]. BAT is localized in the cervical and supraclavicular regions [22, 23]. Only two studies, including ours, have focused on BAT activity during breast cancer chemotherapy [24, 25]. Rousseau et al. studied BAT uptake variations among 33 early breast cancer patients who had 5 FDG PET scans during chemotherapy. The authors found that BAT uptake was highly variable across patients, independently from outdoor temperatures. They also showed that patients treated with taxane-based chemotherapy were those with the more significant changes on PET-CT scan compared to those treated with anthracycline-based chemotherapy [25]. Our team hypothesised that chemotherapy decreases BAT activity and leads to weight gain. Indeed, our previous pilot study on a small sample of patients (26 early breast cancer patients included in the AVATAXHER trial in Jean PERRIN Comprehensive Cancer Centre) showed a decrease in ^18^F-FDG uptake in BAT regions after one course of chemotherapy [24]. More specifically, our team found that patients gaining weight (> 5% of initial weight) during chemotherapy underwent a significant decrease in BAT activity compared to patients who remained stable or lost weight [24]. One limitation of these two studies is that they were conducted on a few patients only. Data on larger cohorts is needed. The primary objective of the present study was to assess the impact of one course of chemotherapy on BAT activity in 109 early breast cancer patients. The secondary objectives were to assess the relationship between BAT activity variations and weight variations at the end of chemotherapy, and to study the factors influencing BAT activity.

## Methods

### Study population and clinical data

The present study was an ancillary analysis to a national prospective multicentre trial, the AVATAXHER trial (NCT01142778), approved by the local ethics committee and the competent authority. A non-opposition letter has been delivered to the patients in order to inform them about the research. Among the 128 patients potentially eligible for our ancillary study, 109 were included in the assessment (there were 2 oppositions to the use of medical data and 17 had missing or uninterpretable PET/CT).

All patients had been diagnosed with HER2-positive early breast cancer and received neoadjuvant chemotherapy (2 cycles of docetaxel + trastuzumab, then patients with a decrease in SUV (< 70%) were randomized in a 2:1 ratio to receive trastuzumab + docetaxel ± bevacizumab for cycles 3 to 6 whereas patients with a change in SUV ≥70% received trastuzumab + docetaxel). Patients were treated with 6 courses of neoadjuvant chemotherapy followed by 12 injections of trastuzumab, one of which was before surgery.

### PET-CT scan review

A specific procedure for the conduct of PET-CT scans is in force in all facilities concerned. Patients were instructed to fast at least 6 h before ^18^F-FDG injection and to avoid muscular effort the day before. After ^18^F-FDG administration, patients were asked to keep still and warm to avoid brown adipose tissue fixation. The temperature in the examination room was the same in each centre.

**Fig. 1:**
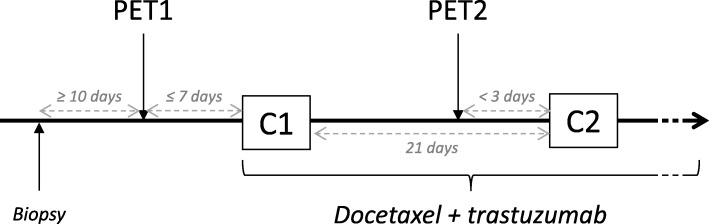
Study design. The first PET scan was realised within 7 days before the first cycle of chemotherapy (C1). The second PET scan was realised less than 3 days before the second cycle of chemotherapy (C2). PET: Positron emission tomography; C1/2: cycle 1/2

Early response to treatment was evaluated by fluorine-18 fluorodeoxyglucose (^18^F-FDG) PET/CT scan before (PET 1) and after one course of chemotherapy (PET 2) (Fig. 1). All patients received a short corticosteroid therapy (24 to 48 h), as part as premedication for docetaxel, which must not influenced BAT activity. Indeed, PET1 and PET2 have been realised before any steroid therapy [26] (see Additional file 1). PET-CT images were visualised on Oasis software V1.8.3 (Segami). All the ^18^F-FDG PET/CT scans were centralised and reviewed twice, by two physicians from our institution, experienced in nuclear medicine. The metabolic activity of BAT was measured by the maximum standard uptake value (SUVmax) [27]. A spherical region of interest (ROI) of 10 mm in diameter was used to quantify ^18^F-FDG uptake (maximum standardized uptake value (SUV_max_)) in the BAT regions, i.e. the cervical and supraclavicular regions [28], and in control tissues. ROIs were placed manually on each image in different specific anatomical regions: the contralateral breast (white fat), the deltoid (muscle) and the liver as controls, and the supraclavicular region (right and left), and in the upper and lower cervical regions (right and left).

### Statistical analysis

R software (version 3.5, R-Project, GNU GPL) was used to perform the statistical analyses. Patient characteristics were described using mean and standard deviation or median and range in case of a non-Gaussian distribution for quantitative parameters. For the hypothesis tests, the significance threshold was fixed at 0.05. For comparisons of before/after measures, we used Wilcoxon’s signed-rank test or paired Student’s t-tests. Confidence intervals were based on hypothesis-testing using these two tests. For comparisons between the groups of relative weight variation, we used ANOVA, Welch’s ANOVA or the Kruskal-Wallis’ test (with the Tukey-Kramer method, Dunn’s test with Holm correction or the Games-Howell test in a post-hoc analysis). A subgroup analysis was also conducted on the relative weight subgroups, without multiple testing corrections. It is worth noting that, for the main objective (before/after difference in SUVmax), with 109 patients it can be thought that our study had enough power (> 85%) to detect an effect size of 0.3 (paired t-test, α = 0.05). Similarly, for the secondary objective of finding a correlation between the weight variation and the SUVmax variation, the power to detect a coefficient equal to 0.3 can be expected to reach 80%.

## Results

### Patient characteristics

The characteristics of the 109 women included in this study are presented in Table 1. The patients’ median age was 48 years. Before chemotherapy, their mean BMI was 25.1 kg/m^2^ (SD 5.7), 25.7% (*n* = 28) of the population were overweight and 13.8% (*n* = 15) were obese. The majority of the PET scans occurred in warm seasons, with 43.1% (*n* = 47) PET scans performed in summer, 19.3% (*n* = 21) in spring and 20.2% (*n* = 22) in autumn and 17.4% (*n* = 19) in winter. All patients are maintained euthyroid. Eight patients were under beta-blockers at PET 1 (before chemotherapy) but were not excluded from the analyses because this does not interfere with SUVmax variation after one course of chemotherapy.

**Table 1:** Patient characteristics according to subgroup of weight variation during chemotherapy. (p represents the *p*-value based on ANOVA, Welch’s ANOVA, or Kruskal-Wallis test for continuous data, and the Chi-squared test for categorical data)

	Overall		GAIN		STABLE		LOSS		*p*
Overall									
	n		n		n		n		
Median age (range) — yrs	109	48 (25–74)	32	48 (26–62)	66	49 (25–74)	11	61 (40–70)	0.024
Mean weight (SD) — kg									
At baseline	109	66.5 (15.2)	32	60.2 (8.9)	66	67.1 (14.8)	11	81.5 (21.5)	0.01
At the end of chemotherapy	109	67.5 (14)	32	65.3 (9.8)	66	67.5 (14.6)	11	73.8 (19.6)	0.686
At the end of Herceptin®	109	67.1 (14.5)	32	63.9 (9.7)	61	67.6 (15.1)	10	73.8 (21.3)	0.212
BMI (SD) — kg/m^2^									
At baseline	109	25.1 (5.7)	32	22.6 (3.7)	66	25.3 (5.2)	11	31.2 (8.4)	0.005
At the end of chemotherapy	109	25.5 (5.3)	32	24.5 (4.1)	66	25.5 (5.2)	11	28.3 (7.8)	0.579
At the end of Herceptin®	109	25.3 (5.5)	32	24.0 (4.1)	61	25.5 (5.4)	10	28.5 (8.5)	0.148
Contraceptive status — %									0.203
Yes	52	47.7	19	59.4	30	45.5	3	27.3	
No	10	9.2	3	9.4	6	9.1	1	9.1	
Sterile	12	11	4	12.5	8	12.1	0	0	
Menopaused	35	32.1	6	18.8	22	33.3	7	63.6	
Tumor — %									
pT									0.078
T2	84	77.1	23	71.9	55	83.3	6	54.5	
T3	25	22.9	9	28.1	11	16.7	5	45.5	
pN									0.048
N0	50	45.9	9	28.1	36	54.5	5	45.5	
N1	59	54.1	23	71.9	30	45.5	6	54.5	
M									0.745
M0	105	96.3	31	96.9	63	95.5	11	100	
Mx	4	3.7	1	3.1	3	4.5	0	0	
SBR grade									0.36
I	1	0.9	1	3.1	0	0	0	0	
II	50	45.9	11	34.4	33	50	6	54.5	
III	56	51.4	19	59.4	32	48.5	5	45.5	
Unknown	2	1.8	1	3.1	1	1.5	0	0	
IHC results									
Overexpressed	106	97.2	32	100	63	95.5	11	100	
Non-determined	3	2.8	0	0	3	4.5	0	0	
Treatment — %									
Docetaxel + Trastuzumab + Bevacizumab	35	32.1	7	21.9	23	34.8	5	45.5	
Docetaxel + Trastuzumab	74	67.9	25	78.1	43	65.2	6	54.5	

### Weight variation

Patient weight did not change between PET 1 and PET 2 because of the short period between these two images. In the overall population, the mean weight change from baseline to the end of neoadjuvant chemotherapy was 1.5 kg (95% CI [0.5, 2.3], *p* = 0.001), or, in relative difference, 1.9% (95% CI [0.8, 3.02], *p* = 0.001): 10.1% (*n* = 11) of the patients lost more than 5% of their initial weight (LOSS subgroup) and 29.4% (*n* = 32) gained more than 5% of their initial weight (GAIN subgroup), while 60.6% (*n* = 66) remained stable.

For the entire period of treatment (from baseline to the last administration of trastuzumab), the mean weight change for the overall population was a gain of 1.2 kg (95% CI [0.29, 2], *p* = 0.011), or, in relative difference, 2.08% (95% CI [0.56, 3.35], *p* = 0.007).

The treatment arm was not linked to weight variation, whether during neoadjuvant treatment (*p* = 0.147) or after the end of the overall period of treatment (*p* = 0.637).

There were significant differences between the GAIN and LOSS patient subgroups (Table 1). At baseline, patients who lost weight were older compared to those who gained weight (*p* = 0.018) and had a higher initial BMI (31.2 kg/m^2^ (SD 8.4) against 22.6 kg/m^2^ (SD 3.7) for the GAIN patient subgroup) (*p* = 0.01). Finally, there was more axillary node involvement in the GAIN patient subgroup compared to the STABLE and LOSS subgroups (*p* = 0.029).

### ^18^F-FDG uptake variation after one course of chemotherapy

Overall, after one course of chemotherapy, ^18^F-FDG uptake in the BAT regions decreased (weak statistical significance) by 0.05 (95% CI [− 0.1, − 0.01], *p* = 0.022), or, in relative difference, by 4.7% (*p* = 0.056).

After one course of chemotherapy, BAT activity decreased for 57% (*n* = 62) of the patients and increased for 43% (*n* = 47). No variation in ^18^F-FDG uptake in the muscle and liver control measures was observed. However, in contralateral breast white adipose tissue there was a decrease of weak statistical significance in SUV_max_ (− 0.02; 95% CI [− 0.03; 0]; *p* = 0.043).

### Factor influencing ^18^F-FDG uptake and the association between weight variation and ^18^F-FDG uptake

The mean initial ^18^F-FDG uptake (PET1: before chemotherapy) in the BAT regions was correlated with initial weight but was independent from age, initial BMI and season. Although the mean initial SUV_max_ (supraclavicular + cervical) was not statistically significantly different between subgroups, the supraclavicular initial SUV_max_ was significantly higher in the GAIN subgroup than in the LOSS subgroup (right side: *p* = 0.006; left side: *p* < 0.001).

^18^F-FDG uptake in the BAT regions after one course of chemotherapy was not correlated with BMI (*p* = 0.078), age (*p* = 0.7) or season (*p* = 0.51).

The GAIN patient subgroup underwent a significant decrease in ^18^F-FDG after one course of treatment, with a 13.4% reduction (PET1: 1.31 (SD 1) versus PET2: 0.97 (SD 0.87); *p* = 0.042) (Table 2). Among the GAIN subgroup patients, 69% (*n* = 22) had a decrease in BAT activity. Patients who lost weight and those who remained stable had no significant variation in ^18^F-FDG uptake. Overall, there was no significant relationship between SUVmax variation and weight variation (*p* = 0.181).

**Table 2:** ^18^F-FDG intensity (SUVmax) variation after one course of chemotherapy. (on lines 4 and 6, p represents the p-value based on Wilcoxon’s signed-rank test or the paired Student’s t-test for before/after variation; in the last column, p is the p-value based on Welch’s ANOVA or Kruskal-Wallis test for the differences across the three groups of weight variation)

		Overall population (*n* = 109)	GAIN (*n* = 32)	STABLE (*n* = 66)	LOSS (*n* = 11)	*p*
SUVmax before: mean (SD)		1.16 (0.88)	1.31 (1)	1.14 (0.86)	0.86 (0.38)	*0.201*
SUVmax after: mean (SD)		1.02 (0.9)	0.97 (0.87)	1.08 (0.98)	0.8 (0.24)	*0.537*
ΔSUVmax (SD)	kg	- 0.14 (0.85)	- 0.34 (0.98)	- 0.06 (0.85)	- 0.05 (0.23)	*0.322*
	*p*	*0.022*	*0.022*	*0.254*	*0.898*	
	%	*−4.38 (34.07)*	- 13.42 (35.82)	−0.35 (34.45)	−2.27 (21.6)	*0.28*
	*p*	*0.056*	*0.042*	*0.399*	*0.735*	

## Discussion

This study is the first to show the impact of one course of chemotherapy on BAT activity in a large population of breast cancer patients. The patients who gained weight (> 5% of initial weight) during neoadjuvant chemotherapy were those who underwent a significant decrease in BAT activity. This study confirms the results of the previous pilot study conducted by our team, suggesting that chemotherapy induces a decrease in BAT activity particularly for patients who undergone weight gain [24]. Among the factors potentially implicated in BAT activity variation that were tested in our analyses (age, initial weight, BMI, season), none was found to be statistically significant. Thus, chemotherapy seems to be the only factor affecting BAT activity.

Weight variation is commonly observed among breast cancer patients in the year following diagnosis [7, 29–36]. In the present study, 29.4% of the patients gained weight and 10.1% lost weight. This is consistent with previous results. It has been shown that during different types of chemotherapy (using the threshold of 5%), 25% of the patients gained weight, 10% lost weight and 65% remained stable [37]. In addition, patients treated with chemotherapy involving trastuzumab exhibited greater weight gain than patients treated without trastuzumab. The authors suggested that the reason was the longer duration of regimens including trastuzumab [37]. BAT activity variation could be implicated in weight variation. Gadéa et al. in a study on 26 breast cancer patients showed that patients who gained weight (after 6 courses of neoadjuvant taxane-based chemotherapy) had a significant decrease in BAT activity after the first course of treatment [24].

A recent retrospective study conducted on cancer patients (*n* = 37) compared body fat mass in patients with and without ^18^F-FDG uptake [38]. The authors showed that the presence of BAT activity was associated with a low ratio of abdominal fat to total fat, and alongside less abdominal obesity. According to the literature data, it could be hypothesised that a decrease in BAT activity, associated with later weight gain, could correspond to a gain in abdominal fat mass. It has been shown that hibernoma resection (a benign tumor histologically similar to brown adipose tissue) is associated with an increase in fat mass gain (mainly abdominal) [39]. However, it is well known that the excess of abdominal adipose tissue is a factor for poor prognosis because of its role in different metabolic pathway disturbances (insulin resistance, adipokines, etc.) and oestrogen production.

The present study has some limitations. The specific procedure for the conduct of PET-CT scans is not ideal for the aim of our ancillary study (designed to avoid brown adipose tissue activation). It would have been interesting to assess the evolution of the patients’ body composition to understand the fat and lean body mass changes potentially induced by BAT variations. Moreover, the information about hormone receptor status could have been correlated with these body mass changes and prognosis. Currently, the realisation of early PET-scan routinely is not a recommendation and medico-economic studies will be required to assess feasibility. Investigations on the impact of BAT on energy imbalance, and particularly on energy intake, would also be an interesting perspective. Furthermore, the study of long-term survival rates could provide information on the impact of such changes on prognosis.

## Conclusion

To the best of our knowledge, this study is the first in a cohort of this type to evidence that one course of chemotherapy induces a decrease in BAT activity among breast cancer patients. Moreover, the patients gaining weight (> 5% of initial weight) were those who underwent the greatest BAT activity decrease compared to those who were stable or who lost weight. Further studies are warranted in order to define the relationship between chemotherapy and BAT activity more precisely. It would also be very interesting to study the evolution of body composition according to BAT activity among breast cancer patients treated with chemotherapy, and its potential impact on survival.

## Supplementary information

**Additional file 1:** [18F]-fluorodeoxyglucose (FDG) positron emission tomography (PET) procedures (adapted from AVATAXHER trial)

## Data Availability

The data used in this study are available from the AVATAXHER study database. However, data are only available for the authors due to the legislation of data protection.

## Abbreviations

^18^F-FDG: Fluorine-18 fluorodeoxyglucose
BAT: Brown adipose tissue
BMI: Body mass index
HER2: Human epidermal growth factor receptor 2
PET-CT: Positron emission tomography-compute tomography
REE: Resting energy expenditure
ROI: Region of interest
SUV_max_: Maximum standardized uptake value
UCP1: Uncoupling protein-1
