# Supplementary material for: A decrease in brown adipose tissue activity is associated with weight gain during chemotherapy in early breast cancer patients
Source: BMC Cancer. 2020 Feb 4;20:96. doi: 10.1186/s12885-020-6591-3 (PMC7001369; doi:10.1186/s12885-020-6591-3)
Supplement: Supplementary file 1 — Additional file 1: [18F]-fluorodeoxyglucose (FDG) positron emission tomography (PET) procedures (adapted from AVATAXHER trial) [file 12885_2020_6591_MOESM1_ESM.pdf]

**[18F]-fluorodeoxyglucose (FDG) positron emission tomography (PET) procedures (adapted from AVATAXHER trial)**

- A common protocol was established to minimise procedure variations between centres.
- PET1 was to be performed ≥10 days after the diagnostic biopsy and ≤7 days before the first course of therapy. PET1 comprised two acquisitions: a total body acquisition followed by a second acquisition focused on the mammary region. PET2 focused on the mammary region only and was to be performed just before the second course of therapy, outside of any corticosteroid administration related to docetaxel perfusion. Patients were withdrawn if the uptake of [18F]-FDG was low (an SUV of ≤ contralateral mammary parenchyma) or if mediastinal lymph node metastases were detected.
- Patient preparation: Avoidance of any muscular/sporting effort during the day before the PET exam.

  No smoking and no nicotine gum. Fasting ≥ 6 hours before PET (no glucose absorption solid or liquid).

  Constant and important hydration. Micturition before starting PET. A venous catheter was installed on the contralateral arm and a 500 mL physiological serum perfusion was performed (utilisation of a central catheter was avoided). Capillary glycaemia was used to exclude hyperglycaemic patients (>11 mmol/L). The plasmatic glycaemia sampled just before [18F]-FDG administration was used for SUV correction. Plasmatic glycaemia was used for SUV correction in cases where capillary glycaemia was absent.
- According to the PET installation, 2–5 MBq/kg [18F]-FDG was IV injected. Patients rested during the 60–80 minute uptake phase in a peaceful and warmed atmosphere to prevent [18F]-FDG brown adipose tissue accumulation. Weight, height, glycaemia, administered dose, injection site, and time delay between [18F]-FDG administration and acquisition were all recorded.
- PET 1: The mammary acquisition had to start imperatively 90 minutes after [18F]-FDG injection. It was centred on the breasts and axillary zones and the patients were asked to adopt the “procubitus” position, with the arms over the head. A dedicated breast mattress was used. Acquisition length was 10 minutes with two 4 minute steps. The total body acquisition was scheduled before or after the mammary acquisition but mammary acquisition had to use the 90 minute delay. The patient was in “decubitus” position, with the arms over the head. The total length of the procedure was variable according to the [18F]-FDG dosage. Each step lasted ≥2 minutes with a total acquisition length of 35 minutes.
- PET 2: Weight and plasmatic glycaemia were re-determined to allow a calculation of [18F]-FDG activity/kg that was identical to that used previously. Mammary acquisition was performed using the same rules as PET1 with the same delay between [18F]-FDG administration and scan acquisition.

- Each centre had to demonstrate that, ≤3 months before starting the study, SUV camera calibration had been performed according the National Electric Manufacturers Association (NEMA) standards and the manufacturer recommendations.
- Data were collected and built according to a camera-specific iterative algorithm to obtain coronal,sagittal, trans-axial and maximum intensity projection images. Collected data were anonymised and saved on a compact disk-read only memory (CD-ROM) in a digital imaging and communications in medicine (DICOM) export format.
